# Adapting and Implementing an Evidence-Based Sun-Safety Education Program in Rural Idaho, 2012

**DOI:** 10.5888/pcd11.130268

**Published:** 2014-05-08

**Authors:** Charlene Cariou, Melanie Gonzales, Hope Krebill

**Affiliations:** Author Affiliations: Melanie Gonzales, St. Luke’s Mountain States Tumor Institute, Twin Falls, Idaho; Hope Krebill, Midwest Cancer Alliance, University of Kansas Medical Center, Kansas City, Kansas.

## Abstract

**Background:**

Melanoma incidence and mortality rates in Idaho are higher than national averages. The importance of increased awareness of skin cancer has been cited by state and local organizations. St. Luke’s Mountain States Tumor Institute (MSTI) prioritized educational outreach efforts to focus on the implementation of a skin cancer prevention program in rural Idaho.

**Community Context:**

As a community cancer center, MSTI expanded cancer education services to include dedicated support to rural communities. Through this expansion, an MSTI educator sought to partner with a community organization to provide sun-safety education. MSTI selected, adapted, and implemented an evidence-based program, Pool Cool.

**Methods:**

The education program was implemented in 5 phases. In Phase I, we identified and recruited a community partner; in Phase 2, after thorough research, we selected a program, Pool Cool; in Phase 3, we planned the details of the program, including identification of desired short- and long-term outcomes and adaptation of existing program materials; in Phase 4, we implemented the program in summer 2012; in Phase 5, we assessed program sustainability and expansion.

**Outcome:**

MSTI developed a sustainable partnership with Payette Municipal Pool, and in summer 2012, we implemented Pool Cool. Sun-safety education was provided to more than 700 young people aged 2 to 17 years, and educational signage and sunscreen benefitted hundreds of additional pool patrons.

**Interpretation:**

Community cancer centers are increasingly being asked to assess community needs and implement evidence-based prevention and screening programs. Clinical staff may become facilitators of evidence-based public health programs. Challenges of implementing evidence-based programs in the context of a community cancer centers are staffing, leveraging of resources, and ongoing training and support.

## Background

Melanoma incidence and mortality rates in Idaho are higher than national rates; thus, skin cancer prevention education has been cited as both a regional and local priority (1). This priority is relevant because knowledge of opportunities to prevent skin cancer is at a low level in this region (2). Certain demographic characteristics, such as education level, appear to predict a person’s uncertainty about cancer prevention activities better than other sociodemographic characteristics (3). The Comprehensive Cancer Alliance for Idaho has prioritized the reduction of sunburns among adults and the reduction of exposure to ultraviolet light among young people (4).

Community outreach and engagement to implement evidence-based programs has been studied in the context of academic and community partnerships (5–7). Increasingly, community clinical health care organizations, including cancer centers, are emphasizing community outreach in the absence of support from academic–community partnerships. The Commission on Cancer of the American College of Surgeons accreditation requirements for 2012 include the following provision: “at least 1 cancer prevention program that is targeted to meet the needs of the community and should be designed to reduce the incidence of a specific cancer type. The prevention program is consistent with evidence-based national guidelines for cancer prevention” (8).

St. Luke’s Mountain States Tumor Institute (MSTI), a 2010–2012 participant in the National Cancer Institute’s Community Cancer Centers Program (NCCCP), determined through a review of the literature that the best approach to skin cancer control was to implement an evidence-based intervention focusing on prevention in an outdoor-based recreational setting (9) and that a community partnership was needed to achieve local support for the program.

## Community Context

St. Luke’s Health System is the only nonprofit health system in Idaho. It is an active member of the communities it serves, with local physicians and boards who further the organization’s mission “to improve the health of people in our region.” MSTI is southern Idaho’s largest provider of cancer services and is nationally recognized by the American Society of Clinical Oncology as a leader in cancer research. Providing advanced care to thousands of patients (3,200 new patients in 2013) at 5 locations across the region, spanning western to south central Idaho, MSTI is also Idaho’s only cancer treatment center for children.

As part of NCCCP, MSTI expanded efforts to achieve the goals of reducing disparities in cancer health care and serving a greater number of medically underserved patients across the full cancer care continuum (5). In 2011, MSTI expanded an existing community cancer education program, consisting of 1 educator in an urban area, to include 2 additional educators and resources and support for the rural communities of southwestern and south central Idaho. On the basis of data on cancer incidence and prevalence, MSTI identified several cancers as focus areas for community outreach: skin, breast, cervical, and colon cancers (1,4). Sun safety and skin cancer education were prioritized because melanoma incidence and mortality rates in Idaho are higher than national rates (1,10). From 2006 through 2010, the incidence of melanoma was 23.1 per 100,000 in Idaho and 19.0 per 100,000 in the United States; in 2009, the annual mortality rate for melanoma was 3.2 per 100,000 in Idaho and 2.7 per 100,000 in the United States (1). Healthy People 2020 set an objective of no more than 2.4 melanoma deaths per 100,000 (10).

The rural community of Payette, Idaho, was selected as the implementation site for a sun-safety educational program because of its community demographics, potential risk factors, and lack of availability of cancer prevention education programs. Payette County, Idaho, is in southwestern Idaho and borders Oregon; it has a population of approximately 22,639; 80.5% are non-Hispanic white, 27.3% are younger than 18 years, and agriculture is a primary form of employment (11). The percentage of adults with a high school education is 83.2%; the state percentage is 88.5% (11). The city sits near the junction of 4 rivers that allow for a variety of outdoor activity.

The Payette Municipal Pool is a hub of activity for the community. It provides recreational opportunities and a convenient location for residents to learn aquatic safety. The facility has a full-sized indoor and outdoor pool, which provides a venue for exercise classes, adult lap swim, regular open swim times, and swim lessons. During the summer, swim lesson enrollment reaches up to 800 young people. Pool users come from both the city of Payette and the surrounding communities.

Availability of cancer prevention programs in Payette is limited. The schools provide education aligned with the curriculum of the Idaho State Department of Education Content Standards for Health: Sun Safety (Box) (12), but other prevention programs that support school efforts are not widely available to the community. There are no local news media in the Payette area. All news media are based in the state capital of Boise; it provides broad messages to the population as a whole and is not tailored to the rural population of Payette.

**Box. Evaluation of Strengths and Limitations (13) of the Idaho State Department of Education Content Standards for Health: Sun Safety**

Strengths:

- Help all students learn more by demanding higher student proficiency and providing effective methods to help students achieve high standards.
- Provide parents, schools, and communities with an unprecedented opportunity to debate and reach agreement.
- Reinforce the best teaching and educational practices already found in classrooms and make them the norm.
- Help the public and local and state educators evaluate which programs work best.

Limitations:

- Vague and unclear standards.
- Careless implementation of standards, and assessment may have negative consequences for students.
- Impose content specifications without taking into account the different needs, opportunities to learn, and skills that may be appropriate for specific districts or regions.

MSTI sought to provide focused sun-safety education to young people aged 2 to 17 years in partnership with a community organization. The implementation of an evidence-based sun-safety program in partnership with the Payette Municipal Pool accomplished the goals of providing a sun-safety program through a sustainable community partnership.The objective of this case study is to describe the process taken by MSTI to engage and partner with a community organization to implement an evidence-based sun-safety program.

## Methods

### Phase 1: Identification and recruitment of a community partner (February 2012)

In 2011, after historically serving only central Idaho urban communities, MSTI expanded an existing community cancer education program to include the rural communities of southwestern and south central Idaho. Before the sun-safety program was selected, potential community partners were identified on the basis of various factors, including population served by the organization, resources available for participation in a mutual educational effort, interest in collaborating on a sun-safety program, and potential for sustainable participation for the organization and MSTI. The MSTI cancer educator applied the principles of community engagement during the identification and selection process (14). These principles, which lead to the formation of a sustainable partnership and program, include developing a purpose and goal for the program, becoming knowledgeable about the community, initiating formal and informal relationships with community leaders, allowing the community to determine the most appropriate intervention for the program location, and building on community assets and strengths (14).

Potential partners were identified by using an asset-mapping strategy. Several organizations were especially attractive as partners because they served young people or engaged young people in outdoor activities. We approached several organizations, including athletic and faith-based organizations, to assess interest in a partnership. The Payette Municipal Pool was identified as the key community partner for southwest Idaho. We selected this organization because of the interest of pool leadership in providing sun-safety education to pool patrons. The Payette Municipal Pool had not only supportive leadership but also a history of providing sun-safety educational inservices to pool staff, in partnership with local dermatologists, and a willingness to collaborate with MSTI and support educational programs through staff time.

### Phase 2: Program selection (March 2012)

The *Guide to Community Preventive Services* highlights the benefits of sun-safety education for adults in recreational settings but identifies inconsistent outcomes for behavior interventions targeted to children (15). We used this information to select a sustainable, evidence-based program from the Research-Tested Intervention Programs database, a National Cancer Institute catalog of evidence-based health intervention programs for cancer prevention and control (16). After multiple sun-safety intervention programs were initially identified, Pool Cool was selected as the best fit because 1) it was evidence-based; 2) the resources and time commitment required to implement the program matched the abilities of MSTI and the Payette Municipal Pool; 3) it was judged to be feasible, sustainable, and potentially expandable. 

The geographical service area of the MSTI community cancer education program ranges over 300 miles across southwestern Idaho and into eastern Oregon. Pool Cool was not only sustainable because of its train-the-trainer model program but it could also be implemented concurrently at multiple pool settings across the MSTI service area.

First implemented in 1999 at pools in Hawaii and Massachusetts, the Pool Cool program is a multicomponent sun-safety education program designed for use at swimming pools (17). Its main objective is to increase sun-protection awareness, motivation, and practices among children aged 5 to10 years who take swim lessons and among parents of the children, pool staff, and other pool users. Initial efficacy trials noted a significantly greater decrease (compared with a control group) in the mean number of sunburns reported by pool staff at facilities who participated in the Pool Cool program (17).

The Pool Cool program teaches children about the risks of overexposure to the sun and encourages them to develop healthy habits for a lifetime. Lessons are taught in conjunction with regular swim lessons, with the Pool Cool curriculum combining education, interactive activities at the pool, and pool-wide environmental changes. The main components are the following:

- A lifeguard/aquatics instructor training module.
- A Leader’s and Decision Maker’s Guidebook for pool staff.
- An 8-lesson curriculum on sun safety presented by aquatics instructors.
- Interactive activities on sun protection conducted for children aged 5 to10 and their parents. Includes demonstrations, games, and puzzles to supplement and support the lessons.
- Provision of shaded areas, signage, and sunscreen dispensers at the pool.
- Promotion of sun-safe environments.

The *Guide to Community Preventive Services* also recommends sun-safety education for recreation staff and access to sunscreen (15).

### Phase 3: Program planning and adaptation (April–June 2012)

The MSTI cancer educator began development of an implementation plan. Initial steps included assessment of resources and activities necessary to implement the program and identification of expected program outputs. Short- and long-term outcomes were also identified in a logic model (Table 1).

**Table 1:** Logic Model for Implementation of Pool Cool Program in Rural Idaho, 2012

Resources	Activities	Outputs	Short- and Long-Term Outcomes	Impact

To accomplish our set of activities we will need the following	To address our problem or asset we will accomplish the following activities	We expect that once accomplished these activities will produce the following evidence or service delivery	We expect that if accomplished these activities will lead to the following changes in 1 to 3 years and 4 to 6 years	We expect that if accomplished these activities will lead to the following changes in 7 to 10 years
Program coordinator. Pool director support. Pool staff (eg, lifeguards, swim instructors). Adaptation of Pool Cool program to meet the needs and branding of MSTI and Payette Municipal Pool. Supplies to facilitate education intervention: leader and decision maker guidebook, laminated lesson cards. Supplies to promote sun-safe behaviors and environment: 3 sunscreen dispensers, sunscreen refills, signage in changing rooms and on fencing. Incentives to staff and students for participating in Pool Cool program.	Obtain support of pool director on any materials to be posted in pool areas (eg, signage, sunscreen dispensers). Conduct training with pool staff; provide detailed overview of Pool Cool lesson plans and supported activities. Install signage and sunscreen dispensers on pool deck and in changing rooms.	Pool environment (signage, availability of sunscreen) will promote a sun-safe environment. Pool staff educated on sun-safe behaviors. Young people enrolled in swim lessons to be educated on sun-safe behaviors (4 Pool Cool rules).	Young people enrolled in swim lessons will know the 4 Pool Cool rules: 1) Protect your skin — use sunscreen. 2) Cover up — after swimming cover your shoulders with a shirt and your legs with long shorts. 3) Protect your face and eyes — wear shades and a hat. 4) Seek shade, limit your time in the sun between 10 am and 4 pm. Pool users/staff will have increased awareness of sun-safe behaviors (from pool education and signage). Increased use of sunscreen during outdoor pool usage (from pool education, signage, and sunscreen availability).	Increased use of sunscreen in community (measured by BRFSS data). Decreased number of sunburns in community (measured by BRFSS data).

Abbreviations: MSTI, Mountain States Tumor Institute; BRFSS, Behavioral Risk Factor Surveillance System.

Although Pool Cool is an existing research-tested program and provides the resources necessary to implement the program, including a Leader’s and Decision Maker’s Guidebook, we needed to adapt the educational and training materials to ensure the program would be feasible for the community partner (Table 2). The guidebook includes information for staff education, sun-safety lesson plans for young people, and other resources. We made the following adaptations to the program: 

**Table 2:** Pool Cool Program Adaptation, Rural Idaho, 2012

Program Component	Action	Program Adjustments
**Leader’s guide**	Adapted	Included MSTI logo and contact information, removed references to activities no longer included in program, provided reference materials, combined Leader’s and Decision Maker’s Guidebook.
**Lifeguard- and instructor-led lessons**	Adapted	Content maintained, added MSTI branding.
**Sun safety signs and sunscreen tips poster**	Adapted, new material created	New educational signage created. Existing verbiage used, images of local pool participants included, MSTI branding and final product reviewed by community partner.
**5 Original poolside activities**	Maintained, removed, or adapted	Weather Watch: the ultraviolet index — maintained.
		Sun Jeopardy — maintained.
		Blue and Purple People — removed because it required additional resources, supplies (eg, colored sunscreens), and time.
		The Emperor’s Clothes — removed because it required additional resources, supplies (eg, assorted clothing items to demonstrate appropriate sun protection), and time.
		Solartech sun exposure card and ultraviolet warning patch/wristband — adapted to provided Pool Cool participants with only an ultraviolet-sensitive card to demonstrate strength of ultraviolet rays.
**Background and reference materials**	Adapted	Removed content and added additional brochures and reference materials: St. Luke’s What you need to know NOW — Skin Cancer Prevention; American Cancer Society — Sun Basics; American Cancer Society — A Parent’s Guide to Skin Protection.
**Sun-safe environment**	Maintained	Shaded areas were already in place at the pool; sunscreen dispensers were added.
**Staff training module**	Developed	Training materials were created with a focus on skin cancer in Idaho, protective factors and risk factors for skin cancer, Pool Cool program overview, and daily sun-safety lessons. Training allowed for time to practice sun safety lessons.

Abbreviation: MSTI, Mountain States Tumor Institute.

- We incorporated organizational branding into the guidebook and revised it to better reflect the activities and lessons that were within the scope of feasibility.
- We eliminated some of the optional poolside activities described by the guidebook and retained the few that were feasible based on available pool resources.
- We adapted print materials and educational signage by revising text and images to increase the number of educational action items and images from the local pool. Signage was reviewed by the community partner; to assess community response before implementation, we asked community members and pool staff to provide feedback on materials.

Additional adaptation included the development of a pool staff training program. MSTI is a regional oncology treatment facility, and its community cancer educators are content experts in cancer prevention and early detection opportunities. As the facilitator of the pool staff training program, the MSTI cancer educator responsible for Pool Cool implementation was a resource for not only the initial train-the-trainer session but also the duration of the Pool Cool program. Staff training for the Pool Cool program included an overview of skin cancer in Idaho, behaviors that increase and decrease cancer risk, and a review of the Pool Cool program and sun-safety lessons. Additional time was provided for pool staff to practice lessons and gain confidence in their ability to teach them. Pool staff completed retrospective posttest training evaluations to assess the benefit of the information provided.

### Phase 4: Program implementation (June–August 2012)

Pool Cool implementation occurred in conjunction with the opening of the outdoor areas of the Payette Municipal Pool in summer 2012. Educational signage and sunscreen dispensers were provided by MSTI, and an initial training session for 13 pool staff members took place in June 2012. An additional training session was provided midway through the summer to orient new staff to the Pool Cool program. Pool Cool lessons were provided to children and adolescents aged 2 through 17 years enrolled in swim lessons from June through August 2012.

### Phase 5: Program sustainability and expansion (August 2012 and ongoing)

MSTI made a long-term commitment to Payette Municipal Pool to provide sun-safety education to young people. Although no formal contract was signed, both MSTI and Payette Municipal Pool are fully invested in sustaining the current role of Pool Cool in swim lessons for young people. MSTI has defined skin cancer prevention as a focused effort for community cancer education as part of NCCCP participation. MSTI intends to expand the Pool Cool program to additional facilities throughout its service area.

With dedicated resources to providing community cancer prevention education, MSTI was able to provide the educational signage, sunscreen dispensers, sunscreen refills, and program incentives to pool staff and swim lesson participants. Additional resources, including staff time for training, sun-safety lessons, and installation of signage and sunscreen dispensers were provided by the Payette Municipal Pool.

## Outcome

The primary objectives of this program were to adapt and implement a sustainable sun-safety education program for young people in a rural community and create a sustainable partnership with a community organization. Through the implementation of Pool Cool at the Payette Municipal Pool, all objectives were met. The partnership between MSTI and the Payette Municipal Pool will be a template for future partnerships for community cancer education initiatives in southwestern Idaho.

During summer 2012, the Pool Cool program served more than 700 young people aged 2 to 17 years enrolled in swim lessons at the Payette Municipal Pool. In addition, hundreds of others benefited from the availability of sunscreen and educational signage at multiple locations throughout the facility. Ultimately, the greatest success was the partnership formed between MSTI and the Payette Municipal Pool. A sustainable partnership was demonstrated through ongoing staff training, commitment to support future programs in MSTI activity planning, and proposed expansion to sites in additional rural communities. Not only was this sustainable partnership formed but MSTI also successfully adapted and implemented its first evidence-based cancer control education program in a community setting.

## Interpretation

The literature has multiple examples of community-engagement activities and evidence-based programs implemented through partnerships between academic institutions and community organizations (5–7,18,19). These relationships are often developed in the context of funded research. To enhance the dissemination of research-tested interventions community organizations organizations are needed to support evidence-based cancer control (20).

Community cancer centers are ideal organizations to support cancer prevention efforts. They are required to provide at least 1 cancer prevention program to maintain accreditation or become accredited by the Commission on Cancer of the American College of Surgeons. Community cancer centers are located throughout the United States in both urban and rural settings, treating 85% of those who have a cancer diagnosis (16). The primary barrier to their efforts in cancer prevention may be staffing. MSTI received National Cancer Institute funding for its community outreach staff. In addition, levels of awareness and use of evidence-based public health may be low among some outreach staff (5,7,19). By leveraging of resources and using the principles of community engagement, community cancer centers can implement evidence-based cancer prevention programs. As noted in *Principles of Community Engagement*, “partnering with the community is necessary to create change and improve health” (14).

Throughout the implementation of Pool Cool, MSTI followed the principles of community engagement (14) and created a sustainable partnership and program. MSTI has demonstrated that by engaging the community, an evidence-based cancer control program can be implemented with limited resources. MSTI will expand the Pool Cool program throughout the region and continue to use the principles of community engagement. The challenge remains in identifying systematic, ongoing support and training to community cancer centers to support their efforts in implementing evidence-based programs with community partners.

## References

[1] National Program of Cancer Registries Cancer Surveillance System. Atlanta (GA): Center for Disease Control and Prevention; 2013. http://statecancerprofiles.cancer.gov/cgi-bin/quickprofiles/profile.pl?16&053. Accessed August 7, 2013.

[2] Health Information National Trends Survey. Bethesda, (MD): National Cancer Institute; 2003. http://hints.cancer.gov/question-details.aspx?dataset=2003&qid=487&qdid=1174. Accessed August 7, 2013.

[3] Health Information National Trends Survey Brief, Number 9. Bethesda, (MD): National Cancer Institue, 2008. http://hints.cancer.gov/docs/HINTS_Brief_9_010708.pdf. Accessed August 7, 2013.

[4] Comprehensive Cancer Alliance for Idaho strategic plan objectives 2011–2015. Boise (ID): Comprehensive Cancer Alliance for Idaho. http://www.healthandwelfare.idaho.gov/Portals/0/Health/Disease/Comp Cancer/CCAI Strategic Plan Objectives Update 07-13.pdf. Updated July 13, 2012. Accessed August 7, 2013.

[5] Clauser SB , Johnson MR , O’Brien DM , Beveridge JM , Fennell ML , Klauzny AD . Improving clinical research and cancer care delivery in community settings: evaluating the NCI community cancer center program. Implement Sci 2009;4:63. 10.1186/1748-5908-4-63 19781094 PMC2764567

[6] Meade CD , Menard JM , Luque JS , Martinez-Tyson D , Gwede CK . Creating community-academic partnerships for cancer disparities research and health promotion. Health Promot Pract 2011;12(3):456–62. 10.1177/1524839909341035 19822724 PMC3653573

[7] Calo WA , Fernandez ME , Rivera M , Diaz EC , Correa-Fernandez V , Pattatuci A , Assessing awareness and use of evidence-based programs for cancer control in Puerto Rico. J Cancer Educ 2012;27(3):486–93. 10.1007/s13187-012-0348-x 22528632 PMC3422596

[8] Cancer program standards 2012, version 1.1: Ensuring patient-centered care. Chicago (IL): Commission on Cancer of the American College of Surgeons; 2012 http://www.facs.org/cancer/coc/programstandards2012.html. Accessed August 7, 2013.

[9] Guide to community preventive services. Preventing skin cancer: education and policy approaches in outdoor recreation settings. Atlanta (GA): Centers for Disease Control and Prevention; 2010. http://www.thecommunityguide.org/cancer/skin/education-policy/outdoorrecreation.html.Updated August 24, 2010. Accessed May 10, 2013.

[10] Healthy people 2020. Washington (DC): US Department of Health and Human Services, Office of Disease Prevention and Health Promotion. http://healthypeople.gov/2020/topicsobjectives2020/objectiveslist.aspx?topicId=5. Accessed August 7, 2013.

[11] State and county quick facts. Washington (DC): US Census Bureau; 2013. http://quickfacts.census.gov/qfd/states/16000.html. Updated June 27, 2013. Accessed August 7, 2013.

[12] Idaho content standards health education. Boise (ID): Idaho State Department of Education, 2010. http://www.sde.idaho.gov/site/csh/docs/Standards/Health Education Standards FINAL Approved by Legislature 1-2010 for Adoption Fall 2010.pdf. Accessed August 7, 2013.

[13] Advantages and disadvantages. http://www.stanford.edu/~hakuta/www/archives/syllabi/CalTex_SBR/procon.html. Accessed December 9, 2013.

[14] Silberberg M , Cook J , Drescher C , McCloskey DJ , Weaver S , Ziegahn L , editors. CTSA Community Engagement Key Function Committee Task Force on the Principles of Community Engagement (2nd edition). Washington (DC): US Department of Health and Human Services, Public Health Service, National Institutes of Health; 2011. p. 45–53.

[15] Guide to Community Preventive Services. Preventing skin cancer: education and policy approaches in outdoor recreation settings. Atlanta (GA): Centers for Disease Control and Prevention; 2010. http://www.thecommunityguide.org/cancer/skin/education-policy/outdoorrecreation.html.

[16] Research-tested interventions programs. Bethesda (MD): National Cancer Institute and Substance Abuse and Mental Health Services Administration. http://rtips.cancer.gov/rtips/. Updated May 21, 2013. Accessed June 2, 2013.

[17] Glanz K , Geller AC , Shigaki D , Maddock JE , Isnee MR . A randomized trial of skin cancer prevention in aquatic settings: the Pool Cool program. Health Psychol 2002;21(6):579–87. 10.1037/0278-6133.21.6.579 12433010

[18] Harrop JP , Nelson DE , Kuratani DG , Dolan Mullen P , Paskett ED . Translating cancer prevention and control research into the community setting: workforce implications. J Cancer Educ 2012;27(Suppl 2):S157–64. 10.1007/s13187-012-0329-0 22362356

[19] Wallerstein N , Duran B. Community-based participatory research contributions to intervention research: the intersection of science and practice to improve health equity. Am J Public Health 2010; 100(Suppl 1): S40–6. 20147663 10.2105/AJPH.2009.184036 PMC2837458

[20] Clinical-community relationships evaluation roadmap. Executive summary. Rockville (MD): Agency for Healthcare Research and Quality. http://www.ahrq.gov/professionals/prevention-chronic-care/resources/clinical-community-relationships-eval-roadmap/ccre-roadmap-summary.html. Updated July 2013. Accessed August 7, 2013.

